# The prevalence of underweight and obesity in Chinese children and adolescents with major depressive disorder and relationship with suicidal ideation and attempted suicide

**DOI:** 10.3389/fpsyt.2023.1130437

**Published:** 2023-05-05

**Authors:** Zhiwei Liu, Liang Sun, Yulong Zhang, Juan Wang, Feng Sun, Zhaokun Zhang, Guangying Sun, Longlong Sun, Rongchun Yang

**Affiliations:** ^1^Department of Psychiatry, The Third People’s Hospital of Fuyang, Fuyang, China; ^2^Department of Psychiatry, Fuyang Mental Health Center, Fuyang, China; ^3^Department of Psychiatry, Chaohu Hospital of Anhui Medical University, Hefei, China; ^4^Department of Psychiatry, School of Mental Health and Psychological Sciences, Anhui Medical University, Hefei, China; ^5^The Clinical Hospital of Chengdu Brain Science Institute, MOE Key Laboratory for Neuroinformation, University of Electronic Science and Technology of China, Chengdu, China; ^6^Department of Psychiatry, The Fourth People’s Hospital of Chengdu, Chengdu, China

**Keywords:** underweight, obesity, suicidal ideation, attempted suicide, children, adolescents, major depressive disorder

## Abstract

**Background:**

The high rates of obesity and suicide have become serious public health problems worldwide, especially in children and adolescents with major depressive disorder (MDD). This research aimed to explore the rates of underweight, overweight or obesity, suicidal ideation and attempted suicide in hospitalized children and adolescents with MDD. Then, we analyzed the correlation between underweight or obesity and suicidal ideation and attempted suicide, and finally obtained the independent influencing factors of underweight or obesity.

**Methods:**

A total of 757 subjects in the Third People’s Hospital of Fuyang from January 2020 to December 2021 were enrolled in this study. According to the underweight, overweight and obesity screening table for school-age children and adolescents published and implemented by the health industry standard of China, all subjects were divided into different body mass index (BMI) categories. We measured fasting blood glucose (FBG) and lipid levels in all subjects and assessed suicidal ideation, attempted suicide, and the severity of depressive symptoms. The socio-demographic and clinical data were collected and analyzed by SPSS 22.0.

**Results:**

The rates of underweight, overweight, obesity, suicidal ideation and attempted suicide were 8.2% (62/757), 15.5% (117/757), 10.4% (79/757), 17.2% (130/757), and 9.9% (75/757), respectively. Correlation analysis indicated that BMIs level was positively correlated with age, age of first hospitalization, total duration of disease, number of hospitalizations, FBG, TG (triglyceride), TC (total cholesterol), LDL (low density lipoprotein), and negatively correlated with HDL (high density lipoprotein). Binary logistic regression analysis showed that male and high level of HDL were risk factors for MDD inpatients with underweight, while high level of TG was a protective factor. Meanwhile, higher levels of FBG, TG and CGI-S were risk factors and suicidal ideation and high dose of antidepressant drugs were protective factors for obesity in children and adolescents with MDD.

**Conclusion:**

The prevalence of underweight, obesity, suicidal ideation and attempted suicide were high in children and adolescents with MDD, and severe depressive symptoms are independent risk factors for obesity, while suicidal ideation and high dose of antidepressants may be protective factors for obesity.

## Introduction

Major depressive disorder (MDD) is a kind of mental illness with high incidence in adolescence. The Chinese national surveys found that the prevalence of MDD in adolescents was about 3.0–23.2% (1, 2). During the COVID-19 pandemic, due to the impact of isolation, school suspension, disrupted pace of life, frequent use of social electronic media and other factors, children and adolescents have significantly increased psychological problems such as depression (3), with a comprehensive prevalence as high as 29% (4). Moreover, the incidence of adolescent obesity increased rapidly during the pandemic (5). It has been reported that the prevalence of underweight, overweight and obesity among Chinese adolescents were 5.9, 10.1 and 5.3%, respectively, and rising continually (6). By 2030, China will rank first among 42 countries with more than one million obese children, followed by India, the United States and Indonesia, according to projections by the World Obesity Federation (7). While the rates of obesity among adolescents have stabilized in developed countries, it continued increasing in low - and middle-income countries (8). Studies have revealed the significant U-shaped association between body mass index (BMI) categories (underweight, normal, overweight and obese) and depressive symptoms (9), suggesting that both underweight and obesity were closely related to severe depressive symptoms (10). Meta-analysis showed that the rates of overweight and obesity in adolescence with depressive disorder were about 9.0–16.9% and 10.1–26.7%, respectively, which were higher than those of healthy people in the same age group (11). Underweight or obesity was closely related to poorer mental health in adolescents (12), and adolescents with weight stigma suffered from heavier depressive symptoms (13). Historical literature has shown a bidirectional association between depressive symptoms and underweight or obesity (14), and depressive symptoms in adolescence could predict underweight or obesity in early or later adulthood (15), and vice versa (16).

Suicide is now the second leading cause of death in adolescents among 10–19 years old, and MDD significantly increases the risk of suicidal ideation, attempted suicide and other self-injury behaviors (17). It has been documented that nearly one in ten high school students reported at least one previous suicide attempt, yet more than 80% of suicide attempts were never recognized by pediatricians (18). Foreign studies have shown that the incidence of suicidal ideation in adolescence population was 13.1% (19). During the COVID-19 epidemic, the rate of suicidal ideation in the general population was 17%, while that in psychiatric patients was 36% (20), and the risk of suicide in the overall population was significantly higher than that before the epidemic (21). According to the Chinese national epidemiological survey, the prevalence of suicidal ideation in adolescents was 15.7%, and both underweight and obesity were associated with a higher risk of suicidal ideation (6). Furthermore, attempted suicide was also significantly associated with depressive symptoms and being underweight or overweight (22). Other studies have shown that BMI level was strongly inversely associated with suicide, and with BMI increasing, the risk of suicide might decrease (23). Attempted suicide was negatively associated with BMI in males. However, in female patients with MDD, there was a U-shaped correlation between attempted suicide and BMI levels (24), suggesting that the risk of attempted suicide was increased in both underweight and obese women. In summary, MDD and suicide-related behaviors are both major global public health issues due to their severe consequences.

To our best knowledge, although there are many studies on different BMI categories or suicidal behaviors in children and adolescents, few studies have discussed the association between underweight or obesity and suicidal ideation and attempted suicide in children and adolescents with MDD. This study aims to explore the prevalence of underweight, overweight or obesity, suicidal ideation and attempted suicide in hospitalized children and adolescents with MDD, and to analyze the correlation between underweight or obesity and suicidal ideation and attempted suicide, and finally to obtain the independent influencing factors of underweight or obesity.

## Methods

### Subjects

The subjects of this study were hospitalized children and adolescents with MDD in the Third People’s Hospital of Fuyang from January 2020 to December 2021. Inclusion criteria: (1) 8–18 years old; and (2) meeting the diagnostic criteria of MDD in the fifth edition of Diagnostic and Statistical Manual of Mental Disorders (DSM-V). Exclusion criteria: (1) Patients with other severe mental disorders in accordance with DSM-V (such as schizophrenia, obsessive–compulsive disorder, etc.); (2) complicated with intellectual disability or severe neurological diseases; and (3) complicated with serious physical diseases (such as cardiovascular disease, digestive system disease, respiratory system disease, etc.). The enrolled patients and their guardians agreed to participate in the study after knowing the purpose, process, and related advantages and disadvantages, and signed an informed consent form. This study was approved by the Medical Ethics Committee of the Third People’s Hospital of Fuyang (granted number: [2018]2018-340-10).

### General information and biochemical indicators

#### Height, weight and BMI

The height and weight of the subjects were measured accurately, keeping one decimal place. BMI was calculated as weight (kg)/height (m)^2^. According to the “Malnutrition Screening of School-age Children and Adolescents” issued and implemented by the health industry standard of the People’s Republic of China (25), the BMI levels of children at different ages, such as 8 years old (boys <14.0, girls <13.6), 9 years old (boys <14.1, girls <13.8), 10 years old (boys <14.4, girls <14.0), 11 years old (boys <14.9, girls <14.3), 12 years (boys <15.4, girls <14.7), 13 years old (boys <15.9, girls <15.3), 14 years old (boys <16.4, girls <16.0), 15 years old (boys <16.9, girls <16.6), 16 years old (boys <17.3, girls <17.0), 17 years old (boys <17.7, girls <17.2), 18 years old (boys <17.9, girls <17.3), and the overall patients were divided into underweight group and non-underweight group. According to the “Overweight and Obesity Screening Table for School-age Children and Adolescents” published and implemented by the health industry standard of the People’s Republic of China (26), according to the BMI levels of children at different ages, such as 8 years old (boys ≥19.7, girls ≥19.4), 9 years old (boys ≥20.8, girls ≥20.4), 10 years old (boys ≥21.9, girls ≥21.5), 11 years old (boys ≥23.0, girls ≥22.7), 12 years old (boys ≥24.1, girls ≥23.9), 13 years old (boys ≥25.2, girls ≥25.0), 14 years old (boys ≥26.1, girls ≥25.9), 15 years old boys/girls ≥26.6, 16 years old boys/girls ≥27.1, 17 years old boys/girls ≥27.6, 18 years old boys/girls ≥28.0. All patients were divided into obesity group and non-obesity group, and also see (26) for criteria of normal-weight group or overweight group.

#### Clinical data collection

Clinical electronic medical records and questionnaires were used to collect the general information of the subjects, such as gender, age, and education level. The age of previous onset, age of first hospitalization, number of hospitalizations, total course of disease, whether accompanied by physical diseases, current medication category and therapeutic dose were recorded. The antidepressant dose of each subject was converted to fluoxetine equivalent, and the conversion formula was: fluoxetine 20 mg = citalopram 20 mg = escitalopram 9 mg = paroxetine 17 mg = sertraline 49.3 mg = venlafaxine 74.7 mg = mirtazapine 25.5 mg = fluvoxamine 71.7 mg (27, 28).

#### Biochemical criterion

On the second day after enrollment, fasting blood glucose (FBG), total cholesterol (TC), triglyceride (TG), high density lipoprotein (HDL), low density lipoprotein (LDL) and other hematological indicators were detected in enrolled patients.

#### Suicidal ideation and attempted suicide

Three simple questions were used to assess whether the patient had suicidal ideation, attempt or behavior in the past 3 months (29), which were: (1) “Have you ever seriously considered suicide or killing yourself?”; (2) “Have you ever made detailed plans to kill yourself?”; (3) “Do you really try to kill yourself in your life?.” A “yes” or “no” answer was given to each question.

#### Clinical global impression

Clinical Global Impression of Severity Scale (CGI-S) was used to assess the severity of the disease. Using an 8-point scoring method from 0 to 7 points (30), according to the disease severity of the specific patient compared with other similar patients in the same study, the score corresponds to the disease severity: 0 – no disease; 1 – essentially no disease; 2 – very light; 3 – mild; 4 – moderate; 5 – heavier; 6 – severe; 7 – extremely heavy.

### Statistical analysis

IBM SPSS 22.0 statistical software was used for data analysis. Measurement data conformed to normal distribution and were expressed as ( $\bar{x}\pm s$ ), and One-Way ANOVA was used for comparison between groups. If not, the results were expressed as median M (P25, P75), and Kruskal Wallis Test was used for comparison between groups. Count data were expressed as constituent ratio [n (%)] and compared by Chi-Square Tests. Spearman rank correlation was used to analyze the correlation between overall patients’ BMI, suicidal ideation, attempted suicide and multiple study variables. Socio-demographic and clinical variables with a value of *p* < 0.05 in univariate analysis were included in binary logistic regression analysis to determine the independent influencing factors of underweight or obesity in children and adolescents with MDD, and to obtain the correlation between underweight or obesity and suicidal ideation and attempted suicide. The test level *α* = 0.05.

## Results

### Comparison between different BMI groups of the patients with MDD

A total of 757 children and adolescents with depressive disorder were enrolled in this study, including 28.4% (215/757) males and 71.6% (542/757) females. The average age was 15 (14, 16) years old, the age of first onset was 14 (12, 15) years old, and the duration of illness was 12 (4, 24) months. The prevalence of underweight, normal weight, overweight and obesity in MDD patients was 8.2% (62/757), 65.9% (499/757), 15.5% (117/757), and 10.4% (79/757), respectively. As shown in Table 1, comparison between different BMI groups showed statistically significant differences in age, gender, age of onset, age of first hospitalization, total duration of disease, number of hospitalizations, FBG, TG, TC, HDL, LDL, types of antidepressant medications, and dosage of antidepressants (*p* < 0.05).

**Table 1:** Comparison of general data and biological indexes of underweight group, normal weight group, overweight group and obesity group.

Variable	Underweight (*n* = 62)	Normal weight (*n* = 499)	Overweight (*n* = 117)	Obese (*n* = 79)	F/X^2^/H	*P*
Age (years)	15.27 ± 1.80	15 (14, 16)	15 (14, 16)	14 (13, 16)	11.206	0.011^c^
Gender (%)					22.741	<0.001^b^
Male	33 (53.2)	135 (27.1)	32 (27.4)	15 (19.0)		
Female	29 (46.8)	364 (72.9)	85 (72.6)	64 (81.0)		
Education (%)					9.359	0.154^b^
Primary school	2 (3.2)	37 (7.4)	11 (9.4)	11 (13.9)		
Junior middle school	35 (56.5)	298 (59.7)	64 (54.6)	49 (62.0)		
High school or above	25 (40.3)	164 (21.9)	41 (35.0)	19 (24.1)		
Age of onset (years)	14.11 ± 2.07	14 (13, 15)	13 (12, 14)	12.85 ± 1.96	22.565	<0.001^c^
Age of first admission (years)	14.89 ± 1.92	15 (13, 16)	14 (13, 16)	14 (13, 15)	10.185	0.017^c^
Total course of disease (months)	6 (2.75, 24)	12 (3, 24)	12 (6, 24)	12 (6, 24)	16.564	0.001^c^
Number of hospitalizations (times)	1 (1, 1)	1 (1, 1)	1 (1, 1)	1 (1, 1)	12.362	0.006^c^
BMI	15.83 ± 0.94	19.58 ± 1.89	24.31 ± 1.44	29.48 ± 3.37	814.932	<0.001^a^
Hematological index						
FBG (mmol/L)	4.93 ± 0.72	4.98 ± 0.48	5.06 ± 0.42	5.18 ± 0.62	4.168	0.006^a^
TG (mmol/L)	0.76 (0.62, 0.87)	0.79 (0.61, 1.09)	1.06 (0.73, 1.46)	1.55 ± 0.82	84.734	<0.001^c^
TC (mmol/L)	3.60 ± 0.80	3.66 (3.24, 4.18)	3.90 ± 0.73	4.42 ± 1.02	46.888	<0.001^c^
HDL (mmol/L)	1.57 ± 0.40	1.41 (1.22, 1.65)	1.42 (1.17, 1.61)	1.30 (1.02, 1.53)	20.248	<0.001^c^
LDL (mmol/L)	2.12 ± 0.59	2.09 (1.73, 2.43)	2.25 (1.95, 2.65)	2.31 (1.59, 3.00)	14.914	0.002^c^
Suicidal ideation (%)					3.440	0.329^b^
Yes	14 (22.6)	77 (15.4)	24 (20.5)	15 (19.0)		
No	48 (77.4)	422 (84.6)	93 (79.5)	64 (81.0)		
Suicidal behavior (%)					2.661	0.447^b^
Yes	5 (8.1)	45 (9.0)	14 (12.0)	11 (13.9)		
No	57 (91.9)	454 (91.0)	103 (88.0)	68 (86.1)		
CGI-S	4 (4, 5)	4 (4, 5)	4 (4, 5)	4 (4, 5)	3.559	0.313^c^
Antidepressant medications (%)					17.064	0.009^b^
No	5 (8.1)	59 (11.8)	16 (13.7)	19 (24.1)		
Monotherapy	51 (82.3)	413 (82.8)	97 (82.9)	60 (75.9)		
Polypharmacy	6 (9.7)	27 (5.4)	4 (3.4)	0		
Antidepressant dosage (mg/d)	20.3 (20, 40)	20.3 (20, 40.15)	20.3 (20, 40.6)	20 (6.23, 32.8)	12.370	0.006^c^
Antipsychotic medications (%)					1.266	0.974^b^
No	36 (58.1)	302 (60.5)	72 (61.5)	51 (64.6)		
Monotherapy	25 (40.3)	185 (37.1)	43 (36.8)	27 (34.2)		
Polypharmacy	1 (1.6)	12 (2.4)	2 (1.7)	1 (1.3)		
Chlorpromazine equivalent (mg/d)	185.55 ± 167.05	150 (75, 200)	150 (75, 181.88)	117.90 ± 135.33	5.765	0.124^c^

^a^One-way ANOVA; ^b^Chi-Square Tests; ^c^Kruskal Wallis Test. NSSI, Non-suicidal self-injury; BMI, body mass index; FBG, fasting blood glucose; TG, triglyceride; TC, total cholesterol; HDL, high-density lipoprotein; LDL, low-density lipoprotein; Monotherapy, Taking one kind of antidepressant or antipsychotic medication; Polypharmacy, Taking two kinds of antidepressant or antipsychotic medication.

### Prevalence of suicidal ideation and attempted suicide in overall patients and different BMI groups

The prevalence of suicidal ideation and attempted suicide was 17.2% (130/757) and 9.9% (75/757) in all subjects, respectively. The prevalence of suicidal ideation in underweight, overweight and obese patients were 22.6% (14/62), 20.5% (24/117), and 19.0% (15/79), respectively. The prevalence of attempted suicide in underweight, overweight and obese patients were 8.1% (5/62), 12.0% (14/117), and 13.9% (11/79), respectively. See Table 1.

### Association of demographic data and clinical variables with BMI, suicidal ideation, and attempted suicide

The Spearman correlation coefficient showed that BMIs were positively correlated with age, age of first hospitalization, total duration of disease, number of hospitalizations, FBG, TG, TC, and LDL, and negatively correlated with HDL (*p* < 0.05). Suicidal ideation was positively correlated with gender, total duration of disease, number of hospitalizations, TC, LDL, attempted suicide, CGI-S, antidepressant dosage, antipsychotic medications and chlorpromazine equivalent (*p* < 0.05). Attempted suicide was positively correlated with gender, total duration of disease, number of hospitalizations, TC, LDL, suicidal ideation, CGI-S, antidepressant dosage, and antipsychotic medications, and negatively correlated with education and age of onset (*p* < 0.05). As shown in Table 2.

**Table 2:** Association of demographic data and clinical variables with BMI, suicidal ideation, and attempted suicide.

Variable	BMI		Suicidal ideation		Attempted suicide	
	*r*	*P*	*r*	*P*	*r*	*P*
Age (years)	0.129	<0.001	0.029	0.432	−0.008	0.817
Gender	0.063	0.084	0.085	0.020	0.111	0.002
Education	0.044	0.223	−0.049	0.178	−0.104	0.004
Age of onset (years)	0.028	0.436	−0.037	0.308	−0.085	0.019
Age of first admission (years)	0.106	0.003	0.046	0.202	−0.007	0.845
Total duration of disease (months)	0.173	<0.001	0.146	<0.001	0.132	<0.001
Number of hospitalizations (times)	0.082	0.024	0.149	<0.001	0.197	<0.001
FBG (mmol/L)	0.102	0.005	−0.040	0.276	0.008	0.816
TG (mmol/L)	0.278	<0.001	0.050	0.173	0.060	0.099
TC (mmol/L)	0.193	<0.001	0.098	0.007	0.075	0.038
HDL (mmol/L)	−0.137	<0.001	−0.038	0.295	−0.034	0.347
LDL (mmol/L)	0.136	<0.001	0.146	<0.001	0.091	0.013
Suicidal ideation	0.017	0.650	1		0.728	<0.001
Attempted suicide	0.057	0.114	0.728	<0.001	1	
CGI-S	0.061	0.094	0.702	<0.001	0.560	<0.001
Antidepressant medications	−0.060	0.098	−0.011	0.769	−0.061	0.091
Antidepressant dosage (mg/d)	−0.020	0.588	0.161	<0.001	0.128	0.001
Antipsychotic medications	−0.014	0.700	0.153	<0.001	0.136	<0.001
Chlorpromazine equivalent (mg/d)	0.010	0.850	0.144	0.007	0.078	0.141

### Influencing factors of underweight and obesity in patients with MDD

Using binary logistic regression analysis with underweight (non-underweight = 0, underweight = 1) as the dependent variable, age, gender (assignment: male = 1, female = 2), age of onset, age of first hospitalization, total duration of disease, number of hospitalizations, FBG, TG, TC, HDL, LDL, suicidal ideation (assignment: no = 0, yes = 1), attempted suicide (assignment: no = 0, yes = 1), CGI-S, several types of antidepressants (assignment: none = 0, one type = 1, two types = 2), dosage of antidepressants as independent variable, the results showed that male (*OR* = 4.04, 95%CI: 2.19–7.44, *p* < 0.001), higher levels of HDL (*OR* = 2.68, 95%CI: 1.24–5.76, *p* = 0.012) were risk factors for MDD adolescents with underweight, and higher levels of TG (*OR* = 0.40, 95%CI: 0.17–0.93, *p* = 0.033) was a protective factor. The *R^2^* of the overall model was 0.158. As shown in Table 3.

**Table 3:** Influencing factors for underweight in patients with MDD.

Variables	Unstandardized coefficients		*P*	*OR*	95.0% CI	
	*B*	*S.E.*			Lower	Upper
Age (years)	−0.346	0.386	0.371	0.71	0.33	1.51
Male (ref. Female)	1.396	0.311	<0.001	4.04	2.19	7.44
Age of onset (years)	0.441	0.380	0.246	1.55	0.74	3.27
Age of first admission (years)	−0.074	0.117	0.528	0.93	0.74	1.17
Total course of disease (months)	0.034	0.033	0.298	1.04	0.97	1.10
Number of hospitalizations (times)	0.371	0.287	0.196	1.45	0.83	2.55
FBG (mmol/L)	−0.361	0.321	0.260	0.70	0.37	1.31
TG (mmol/L)	−0.916	0.430	0.033	0.40	0.17	0.93
TC (mmol/L)	−0.297	0.294	0.312	0.74	0.42	1.32
HDL (mmol/L)	0.984	0.391	0.012	2.68	1.24	5.76
LDL (mmol/L)	0.212	0.484	0.661	1.24	0.48	3.19
Suicidal ideation (ref. No)	−1.122	0.685	0.101	0.33	0.09	1.25
Suicidal behavior (ref. No)	−0.517	0.744	0.487	0.60	0.14	2.56
CGI-S	−0.127	0.289	0.662	0.88	0.50	1.55
Antidepressant medications (ref. No)						
Monotherapy	−1.058	1.541	0.492	0.35	0.02	7.11
Polypharmacy	−0.278	1.641	0.866	0.76	0.03	18.88
Antidepressant dosage	−0.003	0.010	0.768	1.00	0.98	1.02

Similarly, binary logistic regression analysis showed that higher levels of FBG (*OR* = 1.83, 95%CI: 1.13–2.96, *p* = 0.013), TG (*OR* = 1.96, 95%CI: 1.30–2.97, *p* = 0.001) and CGI-S (*OR* = 2.00, 95%CI: 1.16–3.44, *p* = 0.013) were risk factors and suicidal ideation (*OR* = 0.17, 95%CI: 0.04–0.76, *p* = 0.021) and higher dosage of antidepressant drugs (*OR* = 0.97, 95%CI: 0.95–0.99, *p* = 0.015) were protective factors for obesity in children with MDD. The *R^2^* of the overall model was 0.224. As shown in Table 4.

**Table 4:** Influencing factors for obesity in patients with MDD.

Variables	Unstandardized coefficients		*P*	*OR*	95.0% CI	
	*B*	*S.E.*			Lower	Upper
Age (years)	−0.243	0.416	0.560	0.78	0.35	1.77
Male (ref. Female)	−0.509	0.384	0.186	0.60	0.28	1.28
Age of onset (years)	0.085	0.399	0.830	1.09	0.50	2.38
Age of first admission (years)	0.042	0.172	0.809	1.04	0.74	1.46
Total course of disease (months)	0.024	0.035	0.480	1.03	0.96	1.10
Number of hospitalizations (times)	0.320	0.319	0.316	1.3	0.74	2.58
FBG (mmol/L)	0.605	0.244	0.013	1.83	1.13	2.96
TG (mmol/L)	0.674	0.211	0.001	1.96	1.30	2.97
TC (mmol/L)	0.394	0.268	0.141	1.48	0.88	2.51
HDL (mmol/L)	−0.552	0.508	0.277	0.57	0.21	1.56
LDL (mmol/L)	0.598	0.409	0.144	1.82	0.82	4.05
Suicidal ideation (ref. No)	−1.802	0.781	0.021	0.17	0.04	0.76
Suicidal behavior (ref. No)	0.194	0.833	0.816	1.21	0.24	6.22
CSI-S	0.692	0.277	0.013	2.00	1.16	3.44
Antidepressant medications (ref. No)						
Monotherapy	−0.930	1.191	0.435	0.40	0.04	4.07
Polypharmacy	−19.163	6.265E3	0.998	0.00	0.00	
Antidepressant dosage	−0.030	0.012	0.015	0.97	0.95	0.99

## Discussion

This study aimed to explore the prevalence of underweight, overweight and obesity in children and adolescents’ inpatients with MDD, and analyze the correlation between suicidal ideation, attempted suicide and underweight and obesity. The main results are as follows: (1) The prevalence of underweight, overweight, obesity, suicidal ideation and attempted suicide were 8.2% (62/757), 15.5% (117/757), 10.4% (79/757), 17.2% (130/757), and 9.9% (75/757), respectively. (2) Correlation analysis indicated that BMIs were positively correlated with age, age of first hospitalization, total duration of disease, number of hospitalizations, FBG, TG, TC, LDL, and negatively correlated with HDL. (3) Binary logistic regression analysis revealed that male and higher HDL level were risk factors for MDD inpatients with underweight, while higher TG level was a protective factor. Meanwhile, higher levels of FBG, TG and CGI-S were risk factors and suicidal ideation and higher dosage of antidepressant drugs were protective factors for obesity.

Previous study reported the rates of underweight, overweight and obesity of adolescents in China were 5.9, 10.1, and 5.3%, respectively, and the rates of boys in each weight category were higher than that of girls (6). Clinical studies suggested the higher incidence of overweight and obesity in Chinese adolescents with MDD than that of adolescents without MDD (13.15% vs. 9.88, 11.95% vs. 6.75%) (2). A meta-analysis of 11 studies also found that the prevalence of overweight and obesity in children and adolescents with depressive disorder was approximately 9.0–16.9% and 10.1–26.7%, respectively (11). This study indicated that the rates of underweight, overweight and obesity in children and adolescents with MDD were 8.2, 15.5, and 10.4%, respectively, which were roughly the same as previous studies and significantly higher than that of normal adolescents. Literature indicated that underweight adolescents were more likely comorbid depression (31, 32), and underweight was closely associated with more severe depressive symptoms (9, 12) or more internalization problems (33). In addition, a study of comorbid mental disorders in people of different BMI categories found that underweight boys had higher risk comorbid with depressive disorders (34). In addition to the actual measured BMI, perceived underweight was also strongly associated with higher depressive symptoms in adolescents (35, 36). Moreover, the risk of depression was more than two times higher in people who thought they were underweight than in people perceived normal weight (37), and in fact there was a positive correlation between perceived body weight and actual body weight (35, 38). Besides, large cohort studies have revealed that underweight in adolescence significantly could predict depressive symptoms in adulthood, and depressive symptoms in early life also predicted underweight in men in adulthood, suggesting a bidirectional relationship between underweight and depressive symptoms (16).

Historical literature suggested a bidirectional association between depressive symptoms and obesity (14), and the relation between depressive symptoms and obesity seemed to be stronger than that between obesity and depressive symptoms (39). In previous studies, mental health of obese adolescents was significantly lower than that of normal-weight adolescents (40), and obese adolescents may have higher stress levels, depressive symptoms, and lower psychological resilience (41), which were related to stronger inferiority complex, body dissatisfaction, and impaired self-esteem (42, 43). A systematic review of 17,894 participants found that obese adolescents had an obviously higher incidence of depressive symptoms than non-obese adolescents (21.73% vs. 17.96%) (44). Secondly, studies have shown that women who were heavier in adolescence and gained weight significantly in early adulthood had a significantly higher risk of new onset depression (45). Obesity was a significant predictor of depressive symptoms in children and adolescents aged 7–18 years old (46, 47). In addition, compared to healthy weight adolescents, the level of depression in obese adolescents was significantly higher (48), and the risk of developing depressive disorder in the latter group was increased by 1.85 times or more (11, 49). On the other hand, the risk of obesity in children and adolescents with MDD was increased by 2 times (50), and depressive symptoms were closely related to adolescence obesity (51). Furthermore, depressive symptoms could significantly predict adult overweight or obesity (15), while early identification and reduction of depressive symptoms could effectively prevent the occurrence of obesity (52).

In addition to the relationship between measured body weight and depressive symptoms, adolescents who perceived themselves as overweight had a significantly increased risk of depressive symptoms (36). Compared to adolescents with normal weight perception, adolescents who considered themselves overweight had a higher incidence of depression and significantly poorer mental health (53). In addition, a follow-up study showed that self-perceived overweight in early adolescence predicted symptoms of depression and anxiety one year later (54). At present, the causality of the bidirectional relationship between depressive symptoms and obesity is not clear, but both of them do have negative effects on each other.

We found the rates of suicidal ideation and attempted suicide in children and adolescents with MDD were 17.2 and 9.9%, respectively. A study involving five countries in Southeast Asia showed that the prevalence of suicidal ideation and attempted suicide among middle school students were 11.7 and 2.4%, respectively, (22), while the prevalence of suicidal ideation among Chinese adolescents was 15.7% (6), and suicidal ideation or attempted suicide was significantly related to adolescent depressive symptoms and underweight or overweight (6, 22). Previous study has shown that male BMI level was negatively correlated with suicide attempts, and with every 5 kg/m^2^ increase in BMI, the risk of suicide might decrease 15% (23). The U-shaped correlation between BMI level and attempted suicide in depressed women indicates that underweight or obese was related to the higher risk of attempted suicide (24). Other studies have pointed to attempted suicide as an independent risk factor for overweight in adolescents (55). In addition, suicidal ideation and attempted suicide were significantly correlated with self-perception of being overweight or underweight. People who thought they were underweight or overweight had an elevated risk of suicidal ideation or attempted suicide compared to people who thought they were normal weight (38). However, the results of this study showed that suicidal ideation is a protective factor for obesity in children and adolescents with MDD, which is similar to the results of related studies, and may be related to the severe depressive symptoms, long course of disease, and low daily food intake of the subjects (23, 24).

The results of this study suggested that higher dosage of antidepressants was a protective factor for obesity in children and adolescents with MDD. A meta-analysis of 42 studies showed that (SSRIs) and serotonin and norepinephrine reuptake inhibitors (SNRIs) may improve patients’ body weight in the short term, while the long-term effect remains unclear (56). Moreover, former study has pointed out that the higher body mass index was correlated to selective serotonin reuptake inhibitors (SSRIs) treatment in adolescents (57), and the widespread use of antidepressants may lead to a significantly higher long-term risk of weight gain (58). The latest review indicated that weight change induced by antidepressants involves central and peripheral mechanisms, including histamine, cholinergic, 5-serotonin (5-HT), norepinephrine, dopamine, and peripheral effects (59). However, strong evidence demonstrated that fluoxetine was an antidepressant with the low risk of weight gain, and short-term use usually had no significant effect on body weight, or might reduce body weight in obese patients (60, 61). Because the subjects in this study were adolescents, most of the patients took fluoxetine, which might have no effect on weight gain in the short term or improve the body weight index of the patients. Although the prevalence of overweight or obesity in children and adolescents with MDD is significantly increased and is associated with clinical drug use, there are still few clinical trials evaluating antidepressant drug-induced obesity in children and adolescents with MDD.

Our study has several limitations. First, the cross-sectional nature of this study makes it difficult to determine the causal relationship between being underweight or obese and suicidal ideation or attempted suicide. And it seems that the causal relationship between suicidal ideation, high dose of antidepressants and obesity could not be determined. Second, the questionnaire in this study was mostly subjective, and there was no objective scale to assess the psychiatric symptoms of each subject other than depressive symptoms. Subsequent studies can introduce a variety of clinical assessment scales to track the changes of patients’ disease outcome and psychiatric symptoms, so as to clarify the clear correlation between depressive symptoms, weight change, suicidal ideation and attempted suicide in children and adolescents’ inpatients with MDD.

## Conclusion

The high rates of obesity and suicide in children and adolescents have become serious public health problems worldwide, especially in children and adolescents with MDD. The prevalence of underweight, obesity, suicidal ideation and attempted suicide were high in children and adolescents with MDD, and severe depressive symptoms are independent risk factors for obesity, while suicidal ideation and high dose of antidepressants may be protective factors for obesity. In clinical treatment, the weight changes of patients should be closely detected, and the drugs with less influence on weight should be selected as far as possible. The clinical psychiatric symptoms of patients should be evaluated regularly, and the occurrence of suicidal ideation and attempted suicide should be closely monitored to reduce the occurrence of related adverse events.

## Data availability statement

The raw data supporting the conclusions of this article will be made available by the authors, without undue reservation.

## Ethics statement

The studies involving human participants were reviewed and approved by the Medical Ethics Committee of the Third People’s Hospital of Fuyang. Written informed consent to participate in this study was provided by the participants’ legal guardian/next of kin.

## Author contributions

ZL, LiS, YZ, JW, and RY collected and statistically analyzed the data, and wrote the first draft. All authors contributed to the article and approved the submitted version.

## Funding

This study was funded by grants from Key Medical and Health Specialty Construction Project of Anhui Province, Scientific Research Project of Anhui Provincial Health Commission (No. AHWJ2021a035), and Scientific Research Project of Fuyang Municipal Health Commission (No. FY2021-059).

## Conflict of interest

The authors declare that the research was conducted in the absence of any commercial or financial relationships that could be construed as a potential conflict of interest.

## Publisher’s note

All claims expressed in this article are solely those of the authors and do not necessarily represent those of their affiliated organizations, or those of the publisher, the editors and the reviewers. Any product that may be evaluated in this article, or claim that may be made by its manufacturer, is not guaranteed or endorsed by the publisher.
